# Erythromelalgia? A Clinical Study of People Who Experience Red, Hot, Painful Feet in the Community

**DOI:** 10.1155/2013/864961

**Published:** 2013-05-15

**Authors:** D. Friberg, T. Chen, G. Tarr, A. van Rij

**Affiliations:** Department of Surgical Sciences, Otago Medical School Dunedin, University of Otago, Dunedin 9054, New Zealand

## Abstract

We recruited a population of people who clinically suffer from the symptoms of erythromelalgia, red, hot, painful feet made worse by heat and improved by cooling, to better characterise this population and measure their quality of life (QOL). Ninety-two individuals completed the QOL surveys, and 56 individuals were clinically assessed. There was a 3 : 1 ratio of females to males with an average age of 61 years. The estimated prevalence of people who had clinical symptoms of erythromelalgia in the Dunedin community was 15/100,000. Only 27% of people had received a diagnosis for their symptoms despite seeking medical attention. People in the study population had worse quality of life than the general New Zealand population (*P* < 0.001). In the majority of participants symptoms had a mild-moderate effect on their quality of life. The results of this study indicate that the number of people who have clinical symptoms of erythromelalgia is much greater than is commonly accepted and that the majority of these individuals go unrecognised by the medical profession despite seeking help. They have significantly diminished QOL with the majority of people having mild-to-moderate symptoms.

## 1. Introduction 

Erythromelalgia (EM) defines a clinical syndrome of erythema, increased temperature, and associated discomfort, including burning pain, tingling, or similar sensations, preferentially involving the extremities brought on or aggravated by standing, walking, or heat and relieved by the horizontal position and by cold [1–4]. The symptoms most commonly affect the feet and legs bilaterally but can affect hands and even the face [5]. The condition is also associated with anhidrosis [6]. Erythromelalgia has been documented in the literature for almost 150 years, but its prevalence, aetiology, and pathogenesis remain elusive. Even its nomenclature is complex. Erythromelalgia [1, 2], erythralgia [3], and erythermalgia [4]describe overlapping diseases and are used synonymously by most authors, while other distinguish between them based on underlying disorders. Erythromelalgia here will be used as an overarching term for the symptoms described previously. Erythromelalgia occurs either as a primary or secondary disorder. Secondary EM occurs in association with a number of conditions including small fibre peripheral neuropathy of any cause, for example, diabetes, or secondary to myeloproliferative diseases, mushroom poisoning, as a side effect of some medications or as a paraneoplastic syndrome [5, 7–18]. Those who suffer from primary EM develop symptoms without an identifiable cause. A number of hypotheses have been put forward to explain primary EM including vascular shunting, neuropathic aetiologies, microvascular aetiologies, and inflammatory aetiologies. 

In a rare familial form of EM the onset is at a juvenile age and is due to a genetic mutation in the voltage-gated sodium channel alpha-subunit gene SCN9A (OMIM 603415) [19], that is, selectively expressed in the nervous system within the dorsal root ganglion (DRG) and sympathetic ganglion neurons [20]. This leads to increased excitability in neurons and causes increased burning pain transmission and increased blood flow to the skin [20, 21]. However, primary EM mostly occurs in those over the age of 30, and the percentage of patients reporting a first degree relative with EM symptoms is on average 7% [5, 22–24]. Mutations have been shown to develop in individuals *de novo* [25], but further genetic work needs to be done to adequately explain the pathogenesis of primary EM in individuals who develop symptoms later in life.

Erythromelalgia is widely thought to be a rare condition, even though there is little data on its prevalence. Recent studies suggest that the condition is more common than was once believed and that there is a spectrum of symptom experiences from trivial distress to limb-threatening disease [5, 22]. A study of 87 individuals in the Norwegian population estimated that the incidence is 0.25/100,000 inhabitants per year with a corresponding annual prevalence of 2/100,000 [22]. In a population-based study of EM in Olmsted County, MN, USA, the incidence was estimated to be 1.3 per 100,000 [24]. These studies have all been based on referred patients with presumably more severe manifestations, excluding those with milder symptoms and underestimating the true incidence. This study aimed to recruit a self-identified population of people who suffer from the symptoms of red, hot, painful feet from the Dunedin community in order to gain insight into the prevalence of suffers, better characterise this population, and measure their quality of life (QOL).

## 2. Methods Subject Recruitment and Selection 

Participants were recruited from the community over a period of three months. We advertised for volunteers by placing free flyers outside the ground floor elevators of the Dunedin Public Hospital over a three-month period from December through February 2008/2009. On December 18, 2008, a quarter page advertisement was placed in the free, weekly, community publication, the Star. In the same issue, the newspaper ran a story about our study and the experience of a man who suffered from severe EM. Individuals were invited to contact us by phone or email to participate in the study if they suffered from episodes of painful, red, hot feet.

An initial discussion took place with each participant by phone. The study was explained in further detail, and symptoms and experiences with hot feet were discussed. We included anyone who contacted us provided they suffered from painful burning feet, and lived in New Zealand. Individuals who lived outside of the Dunedin city limits were not included in prevalence calculations but were included in quality of life assessments calculations, which we compared to New Zealand population data and demographic descriptors of the study population. The participants were then mailed a packet containing a return envelope, an instruction and information sheet about the study, a consent form, a SF-36 questionnaire, and a quality of life condition specific questionnaire. At the end of the condition specific questionnaire was a free text comment area, and many people chose to express experiences and ideas about their condition. From this initial population a database was created.

In March and June, 2011, participants were invited by telephone, with the entire list being called in completion on five different days at different times of the day. Participants who were invited to attend our department came into the hospital to complete a standardised questionnaire assessment and examination conducted by the lead author regarding their symptoms and experiences with hot feet to confirm the diagnosis of EM. Diagnosis was made if the patient gave a clear history of symptoms of intermittent, bilateral, erythema, and hot pain/discomfort of the extremities, made worse with heat/limb dependency and better with cooling. Other identifiable clinical causes for these symptoms in history and examination were looked at for exclusion. People were given the opportunity to express experiences and ideas about their condition which were also documented at the end of the questionnaire. 

### 2.1. General and Condition Specific Quality of Life Questionnaire

The Medical Outcomes Study Short Form 36-Questionnaire (SF-36) and the validated Otago Condition Specific Questionnaire (OCSQ) [26–28] tool were used to measure QOL. The OCSQ is a 12-item tool that includes pain, discomfort, difficulty coping, perceived appearance, and fear as caused by the specific condition and impact on work, home life, relationships, mood, and leisure activities. Each question is given equal weight, and the total score is converted to 0–100 scale with 0 being no impact. Demographic information including age, sex, vocation, and residential address was also obtained. Comparison of SF-36 scores was made with the average score and confidence intervals of the general New Zealand population taken from the 2006/07 New Zealand health survey [29]. Individual data from the New Zealand health survey is not available, so it was not possible to age and sex match individuals for our study. The New Zealand health survey does describe scores for smaller populations based on sex and age. We compared scores from the study population to scores of females between the ages of 65–74 years. The reason for this was twofold. Firstly, the hot feet population had on average more females in older age groups than the general New Zealand population. Secondly, the New Zealand health survey showed that females have significantly worse quality of life than males and that older people have significantly worse quality of life than younger people. By choosing scores from an older female population, significant differences are most likely to be explained by hot feet. This is a conservative assumption. The OCSQ scores were compared with the scores of patients with varicose veins who have been assessed using the OCSQ. Varicose veins were selected as a comparison condition because they are a chronic condition that also affects the lower legs and has a wide range of impact on sufferers. To assess the spectrum of severity within our self-identified population, scores from each quality of life measure were divided into five groups. We were interested in whether our self-identified population would include a group of individuals who had mild symptoms, a population that may not have previously come to the attention of the medical community.

### 2.2. Statistics

StatView version 5.01 (SAS Institute) was used to perform statistical analysis. The distribution of continuous variables (kurtosis and skewness) was assessed and analysed with the Mann-Whitney *U*, Student's *t*-test. Raw data for the New Zealand health survey respondents was not available therefore we utilized published means, sample sizes, and confidence intervals (estimating standard error of the mean by SEM = [95%CI/3.92]) for each subscale. The SF36 subscales were then compared via two-tailed *t*-test using an online calculator which allowed input of mean, sample size, and standard error of the mean to derive a *P* value (QuickCalcs, http://www.graphpad.com/quickcalcs/ttest1.cfm?Format=SEM, GraphPad Softward, Inc. 2002–2005). Multiple logistic regression was used to adjust the association of quality of life scores by age and gender. Non-normally distributed variables were log transformed. Residuals were examined for deviance from normal distribution to check that assumptions were met. Results were given as mean ± SD, except non-normally distributed variables, which were expressed as medians and interquartile ranges. A *P* value less than 0.05 was considered statistically significant.

### 2.3. Ethics

This study was approved by the Lower Southern Regional Ethics Committee under application LRS/08/32/EXP.

## 3. Results 

One hundred individuals contacted us about the study. Ninety-seven individuals were mailed a survey. Of the three others one did not live in New Zealand, one did not suffer from hot burning feet, and one decided not to participate. Ninety-two individuals (91%) completed and returned the SF-36 and OCSQ.

From the ninety-two individuals included in the initial quality of life mail out survey, fifty-six individuals completed a face-to-face assessment with the lead author. Of those who did not come forward for a personal interview, eight decided that they no longer wanted to participate, of whom two no longer had symptoms, and one was too unwell to be involved. Eight individuals had scheduling conflicts during the time that the study was undertaken. We were not able to make contact with nineteen of the participants over the recruitment period. One individual gave a clear history of angioedema and had previously received a diagnosis of angioedema and was excluded from the study.

### 3.1. Diagnosis of EM

Of the 56 individuals who attended clinic for assessment, 24 people had strong evidence of EM based on a clear history of intermittent, bilateral, hot pain/discomfort, and redness of the extremities made better with cooling and worse with heat. From history and examination, there was no other clinical diagnosis to explain their symptoms. 

### 3.2. Age

**Figure 1:**
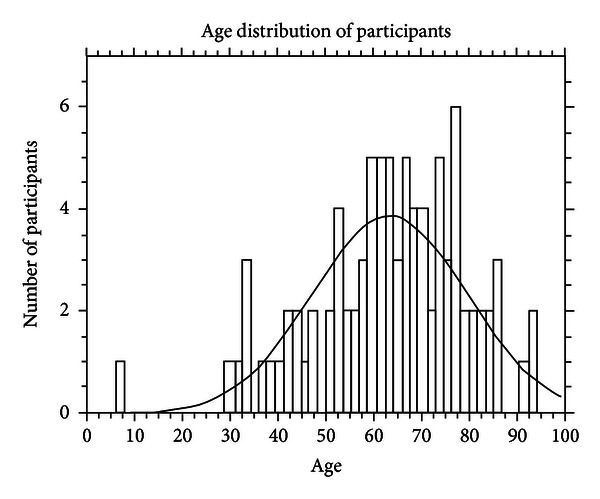
The age distribution of 92 participants who self-identify as having burning hot feet.

Of the 56 individuals who presented for further assessment, the average age was 65 (±18), and the average age of the 36 who did not attend was not different 61 (±14) (*P* = 0.26) (Figure 1).

### 3.3. Sex

Of the population of 92 individuals who self-identified as having red hot feet, 73% were female, and 26% were male. The gender proportions were the same for those who attended the assessments and those who did not (*P* = 0.85). 

### 3.4. Contact with the Medical Profession and Diagnosis

Of the 56 individuals who completed face-to-face assessments, 10 (18%) patients had never talked to their doctor about their symptoms, 42 (75%) had spoken to their primary care doctor, and 20 (36%) participants had spoken to at least one specialist about their symptoms, not necessarily on a visit primarily for their hot feet symptoms. Specialists that patients had spoken to about their condition included: neurologists (11), vascular specialists (4), dermatologists (4), rheumatologists (4) pain specialists (3), palliative care specialists (3), and orthopaedic surgeons (1). Many people commented that their doctors did not know what to make of their symptoms. Two people had been told that they would need psychiatric consultation. Fifteen (27%) patients felt that they had received a diagnosis for their symptoms from a doctor including: erythromelalgia (9), peripheral neuropathy (2), Raynaud's syndrome (1), rheumatoid arthritis (1), restless legs syndrome (1), and peripheral nerve damage caused by electrocution (1).

### 3.5. Symptoms

Based on personal interview, thirty-four (61%) individuals felt that redness was a noticeable part of their symptom complex. Forty-nine (87%) individuals described themselves as having painful feet, and 7 (13%) individuals stated that their symptoms were not more than discomfort. Forty-eight (86%) individuals stated that their feet felt hot during symptomatic episodes. Forty-three (77%) people did not feel that their feet were sweaty during symptomatic episodes. Heat brought on the symptoms in 51 (91%) individuals, whereas cooling improved their symptoms in 53 (94%) of individuals.

### 3.6. Medications

When asked if they had ever tried medications to relief their symptoms 34 (61%) said yes (Table 1).

### 3.7. Relatives

Eighteen participants (32%) said they had a first degree relative with similar symptoms. 

### 3.8. Prevalence

We targeted the city of Dunedin in our recruitment methods, but we had a number of individuals who contacted us from outside the city limits. Of the 24 people who had all five symptoms, 18 lived within the Dunedin city limits. Dunedin has a population of 118,683 inhabitants [30], so the estimated prevalence of individuals who have the full symptom complex of EM would be at least 15/100,000 in Dunedin.

### 3.9. Quality of Life Scores

There were no significant differences in scores on either the SF-36 or Otago condition specific questionnaire between individuals who participated in personal interviews and those who did not. 

### 3.10. SF-36 Global Quality of Life Survey

There was no correlation between test score and age or sex in the self-identified population. When compared to mean scores of females between the ages of 65–74 years from the 2006/07 New Zealand health survey, participants in the study scored significantly lower in seven out of eight domains (Table 2). Participants scored significantly worse in the categories of physical function, bodily pain, general health, vitality, social function, emotional roles, and mental health. They scored similarly to the older female population only in physical roles.

### 3.11. Otago Condition Specific Quality of Life Questionnaire

**Figure 2:**
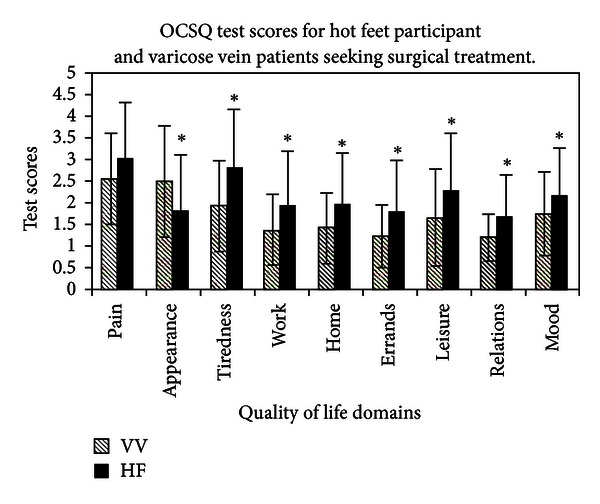
Otago Condition Specific Questionnaire (OCSQ) test scores: VV = varicose veins; HF = hot feet; error bars indicates standard deviations; *indicate significant differences between the varicose vein and hot feet population test scores (*P* < 0.001).

Participant OCSQ scores were compared with previously obtained scores from patients with varicose veins. We had 92 participants with self-identified symptoms of hot feet and 215 participants with varicose veins that completed the OCSQ. The hot feet population was significantly older than the varicose vein population, 62.8 versus 52.4 years (*P* < 0.001). There were more females in the hot feet population at 62.8% versus the varicose vein population 52.4% (*P* = 0.045). After controlling for age and sex, participants scored significantly worse than patients with varicose veins on all questions except for pain, which was similar, and appearance impact that was significantly better (Figure 2).

### 3.12. Spectrum of Symptomatic Effects on QOL

**Figure 3:**
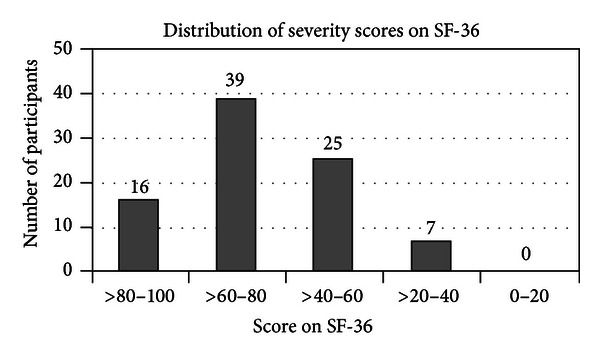
The number of participants that scored within each severity group on the SF-36. Lower scores indicate worse quality of life.

**Figure 4:**
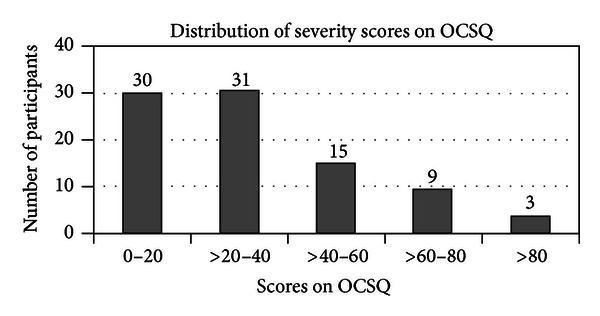
The number of self-identified participants that scored within each severity group on the Otago Condition Specific Questionnaire. Higher scores indicate worse quality of life.

For both the SF-36 and the OCSQ the majority of participants had milder symptoms (Figures 3 and 4).

### 3.13. Qualitative Data

Qualitative data was obtained through comments made. Many people had not received acknowledgment from the medical community regarding their symptoms despite raising it with their community doctor and were surprised and pleased to hear that there might be a name for their condition and that others suffered from it. Another feature was that individuals contacted us from well outside the expected community that was targeted. These people found out about the study through word of mouth.

## 4. Discussion

Historically, erythromelalgia has been thought to be a rare condition but recent evidence indicates that it is probably more common than was once believed and that there is a spectrum of symptom experiences from trivial distress to limb-threatening disease [5, 23]. Of the 56 individuals who completed a personal interview, 24 (42%) individuals clinically had all five characteristics of erythromelalgia: episodes of painful, red, hot feet made better with cooling and worse with heat. Erythromelalgia is a diagnosis often made on clinical history, as patients rarely present during an active flare of their disease, and there are no definitive investigations to determine diagnosis [8]. We estimate the prevalence of individuals suffering from the symptoms of burning feet to be at least 15/100,000 in Dunedin, at least ten times higher than prevalence previously reported [22, 24]. However, unlike all other studies of EM, whose participants are identified through secondary and tertiary referrals, we recruited individuals based on self-identification of symptoms. We expect that our study includes a previously unrecognised group of individuals who suffer from more mild-to-moderate symptoms of red, hot, painful feet consistent with the diagnosis of EM. However, if we include those remaining with most but not all of the diagnostic symptoms of EM, then there is a far greater prevalence of patients with the cluster of symptoms of red hot painful feet.

There was a 1 : 3 male to female ratio in our study population. This is consistent with data from three other large studies [5, 22, 23]. The reason that EM is more common in women is unknown. 

The average participant age was 64.7 years (range 30–94). The average age of patients in the three other large scale studies was 51 years with a range between 5 and 91 years [5, 18, 19]. Our population may be slightly older due to biases in our recruitment methods (hospital and newspaper) that may have preferentially targeted an elderly population. Alternatively milder disease or other overlapping conditions may be more common in the elderly. 

In previous studies individuals who reported a first degree relative with similar symptoms was on average 7% [5, 22–24]. We found a much higher reporting rate of 32%. The reason for this is unclear. A genetic mutation had been identified in families with severe, early onset EM, but we expect that our population contains a larger proportion of individuals with more mild to moderate symptoms. Because many participants came forward through word of mouth, it may be that families with multiple affected individuals were more likely to hear about the study in the community. We had two families with multiple members of the family who participated. 

Participants on average scored worse in 7 out of 8 domains than the respondents of the New Zealand 2006/2007 Health Survey, and it is likely that individuals who suffer from symptoms of red, burning feet have significantly lower quality of life than the general New Zealand population. There was a spectrum of severity of loss of quality of life due to symptoms, with the majority of participants having mild to moderate symptoms. Participants suffered a significantly greater loss of QOL than varicose vein patients seeking treatment through a public health system.

The cause of EM remains unknown, and treatment options largely consist of lifestyle adjustments and pain management. Although it is difficult to treat this condition, it is important to validate the patient's experience. Of the 56 individuals who were personally interviewed, 75% had spoken to a doctor about their condition. Many people commented that their doctors did not know what to make of their symptoms. Two people had been told that they would need psychiatric consultation. Only fifteen (27%) patients felt that they had received a diagnosis for their feet from a doctor. The most significant outcome of this study may be the recognition of a population of individuals who suffer on a daily basis and think that they are the only ones. Many participants were encouraged to know that there was a name for their experience and that others suffered from it. Some had sought recognition from the medical community but were offered none. One man said “I have a bad heart but my feet affect me more on a daily basis. I go to see my doctor on a regular basis for my heart but when it comes to my feet he shrugs his shoulders.” One woman we contacted regarding the (study) said, “she brightened up tremendously, and knowing that there are others out there with the same condition made her sad but happy at the same time because knowing that she was not alone gave her a huge mental boost.” While not all the participants had the full blown features of EM, other obvious clinical causes for their symptoms had been excluded. Other conditions do have symptoms which overlap with those of EM, and their early presence amongst the study subjects cannot be excluded. We would, however, emphasise that these symptoms are more prevalent than often thought and are associated with a significantly diminished quality of life and often go unrecognised by the medical community. Even though EM is now considered primarily to be a neuropathic disorder, these patients attend vascular clinics. It is important that this population is better recognised and characterised to raise awareness and understanding of the condition.

## Figures and Tables

**Table 1:** Percentage of people who have used medications to treat their symptoms and the medications that they could recall that they had tried.

Medications	*n* = 56
Used meds to treat symptoms	34 (61%)
Amitriptyline	12 (32%)
Gabapentin	11 (21%)
Paracetamol	10 (17%)
Sodium valproate	7 (13%)
Aspirin	4 (7%)
Morphine	3 (5%)
Codeine	3 (5%)
Ibuprofen	2 (4%)
Magnesium	2 (4%)
Steroid injection	2 (4%)
Methadone	2 (4%)
Lamotrigine	1 (2%)
Carbamazepine	1 (2%)
Oxynorm	1 (2%)
Epidural	1 (2%)
Dextropropoxyphene	1 (2%)

**Table 2:** The average and confidence interval of SF-36 Global Quality of Life Survey Scores of the self-identified study population compared to female New Zealand health survey respondents between the ages of 65–74 years.

SF-36 domains	Hot feet participants *n* = 92	New Zealand health survey respondents *n* = 460	*P* value
Physical function	60.4 (54.2–66.6)*	72.3 (69.4–75.2)	*P* = 0.0010
Role physical	61.3 (55.1–67.5)	70.3 (66.0–75.2)	*P* = 0.078
Body pain	55.5 (50.2–60.8)*	74.4 (71.6–77.3)	*P* < 0.0001
General health	55.3 (51.1–59.5)*	70.9 (68.7–73.1)	*P* < 0.0001
Vitality	51.3 (48.2–54.4)*	64.9 (62.9–67.0)	*P* < 0.0001
Social function	70.0 (64.0–76.0)*	86.6 (84.5–88.7)	*P* < 0.0001
Role emotional	75.2 (69.6–80.8)*	84.2 (81.0–87.3)	*P* = 0.0006
Mental health	72.8 (67.0–78.3)*	79.1 (77.6–80.6)	*P* = 0.0035

*indicates that hot feet participant average scores were significantly different form the average scores of NZ health survey respondents.
